# Health and Healthy Homes

**Published:** 1880-06

**Authors:** 


HEALTH AND HEALTY HOMES.—A Guide to Domestic Hygiene. By Geo. Wilson, M.A , M.D., Medical Officer of Health for Mid-Warwickshire-Sanitary District, etc. With notes and additions by J. G. Richardson, M.D., Prof. of Hygiene in the University of Pennsylvania, etc. Presley Blakiston, Philadelphia, 1880. Price, $1.50.

Through the popular book store of J. J. & S. P. Richards, we have just received this very useful work. One good feature we notice is the ample table of contents, that points out fully the least detail. General Hygienic Statistics; General Physiology and Pathology of the Human Body; Practical and Scientific Principles of Hygience;

Detailed Considerations on Food and Diet; Prophylaxis and some suggestions in Special Pathology, are some of the most interesting features of the work. Dealing, as it does, not exclusively in hygienic subjects, but also in collateral branches, it forms a book of wide-spread interest, and is calculated to satisfy a general want.

The chapter on "Infectious Diseases and their Prevention," abounds in excellent and practical information and instruction, and together with the one on "Home and its Surroundings," renders the work desirable for a general library.

